# The Role of Vitamin D and Selected Nutrients in the Development of Myopia in Children and Young Adults: A Narrative Review

**DOI:** 10.3390/jcm15103781

**Published:** 2026-05-14

**Authors:** Zuzanna Bomze, Barbara Olędzka, Michał Piątkiewicz, Weronika Dmoch, Piotr Maciejewicz

**Affiliations:** 1 Ophthalmology Student Research Group, Department of Ophthalmology, Medical University of Warsaw, 02-091 Warsaw, Poland; 2 Department and Clinic of Ophthalmology, University Clinical Center, Medical University of Warsaw, 02-091 Warsaw, Poland

**Keywords:** myopia, axial elongation, vitamin D, vitamin A, vitamin E, omega-3 fatty acids, time spent outdoors, children, adolescents

## Abstract

The escalating global prevalence of myopia constitutes a significant public health challenge. This narrative review explores the role of dietary factors, specifically vitamin D and selected nutrients, in its development in children and young adults. Current research underscores a link between low serum vitamin D levels and increased myopia risk. While this association often reflects limited time spent outdoors, vitamin D also appears to exert a direct biological role in regulating ocular growth. The impact of other micronutrients remains ambiguous. Although vitamin A, zinc, and selenium are essential for retinal health and antioxidant defense, human studies regarding their specific capacity to prevent myopia are inconclusive. In contrast, emerging evidence suggests that omega-3 polyunsaturated fatty acids confer protective benefits against axial elongation. Conversely, diets high in refined carbohydrates are associated with an elevated risk of myopia, likely due to insulin-mediated mechanisms influencing scleral structure. Overall, vitamin D and omega-3 fatty acids demonstrate the greatest promise as modifiable protective factors. However, the roles of zinc, selenium, and vitamin A remain inconclusive. Future prevention strategies may benefit from considering nutritional optimization alongside increased outdoor activity as part of a broader strategy of reducing myopia progression.

## 1. Introduction

Myopia is a refractive error characterized by excessive axial elongation of the eye, leading to blurred distance vision [1,2]. It typically manifests in early childhood and progresses during school years, making early life a crucial window for understanding modifiable risk factors and intervention strategies [2]. Environmental and lifestyle factors, in addition to genetic predisposition, have been identified as major contributors to myopia development, with growing evidence highlighting the importance of behaviors such as near-work activities and time spent outdoors [3,4].

Recent findings suggest that the visual environment plays a key role in regulating axial eye growth; however, the strength and independence of these associations remain debated. In a recent longitudinal investigation, it was shown that reduced near-work time combined with increased outdoor exposure was associated with slower axial elongation in children [5]. Similarly, updated meta-analytic evidence suggests that prolonged and intensive near-work is associated with increased risk of myopia and greater axial length, whereas time spent outdoors shows a protective association [6]. Nevertheless, these findings should be interpreted cautiously, as near-work and outdoor exposure are strongly interrelated behaviors, and their effects may not be fully independent. Moreover, heterogeneity in measurement methods (e.g., self-reported vs. objective exposure) and variability in defining near-work intensity limit direct comparability across studies.

Outdoor light exposure is thought to influence ocular growth through retinal signaling pathways and light-dependent biological mechanisms that may slow axial elongation [7,8]. Increased time spent outdoors facilitates distance viewing, which promotes accommodative relaxation, may limit excessive axial elongation of the globe, and consequently reduces the risk of myopia onset and progression. Vitamin D, a fat-soluble secosteroid produced in the skin following ultraviolet B (UVB) exposure and obtained from dietary sources, has been studied in relation to myopia due to its associations with outdoor activity and potential roles in ocular tissue biology [9,10]. Other nutritional factors, including selected micronutrients (e.g., vitamins A, E, and omega-3 fatty acids), have also been investigated, highlighting the multifactorial nature of diet and myopia risk [11,12,13]. However, their role in refractive development remains less clearly defined. Despite growing interest, it remains unclear whether vitamin D represents an independent biological factor in myopia development or primarily reflects outdoor exposure.

**Figure 1:**
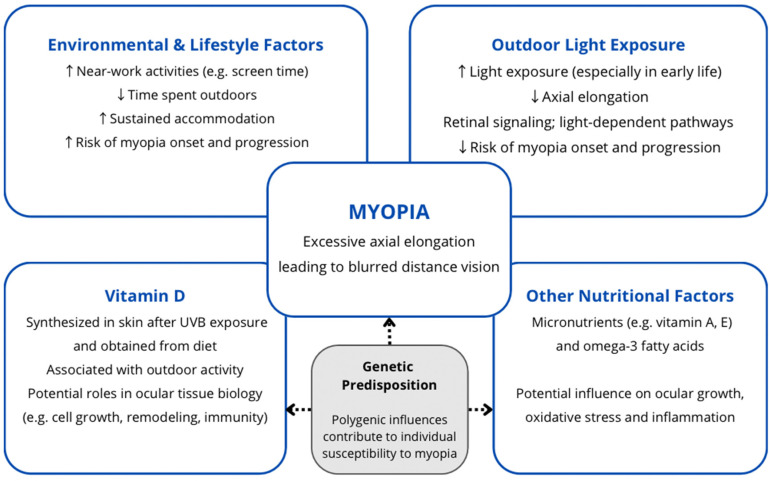
Proposed factors and mechanisms influencing myopia development. Environmental and lifestyle factors, including near-work activities and outdoor time, as well as nutritional factors such as vitamin D and other micronutrients, may affect ocular growth and influence the risk of myopia onset and progression. Genetic predisposition contributes to individual susceptibility.

This narrative review synthesizes evidence published between 2000 and 2025, with particular emphasis on studies from the past decade. Its primary objective is to provide a comprehensive overview of the role of vitamin D and selected nutrients in the onset and progression of myopia in children and young adults. By integrating findings from clinical and epidemiological research, as well as prior review articles, this paper aims to critically evaluate the current body of knowledge, identify inconsistencies and research gaps, and propose directions for future investigations in this area. [Figure 1].

## 2. Methods

This narrative review was conducted using a structured literature search strategy to identify and synthesize current evidence on the role of vitamin D and selected nutrients in the development of myopia in children and young adults. A comprehensive search of the literature was performed in PubMed (National Library of Medicine, Bethesda, MD, USA), Web of Science (Clarivate, London, UK), and Scopus (Elsevier B.V., Amsterdam, The Netherlands) on 15 January 2026.

The search strategy combined Medical Subject Headings (MeSH) and free-text terms related to myopia and nutritional factors. Keywords included: “myopia,” “axial elongation,” “vitamin D,” “25-hydroxyvitamin D,” “vitamin A,” “retinol,” “microelements”, “zinc,” “selenium,” “omega-3 fatty acids,” “polyunsaturated fatty acids,” and “refined carbohydrates,” combined using Boolean operators (AND, OR). An example search strategy used in PubMed was: (“myopia” OR “axial elongation”) AND (“vitamin D” OR “25-hydroxyvitamin D” OR “vitamin A” OR “retinol” OR “zinc” OR “selenium” OR “omega-3 fatty acids” OR “polyunsaturated fatty acids” OR “refined carbohydrates”).

Studies published between 2015 and 2026 in English and involving human participants were considered. This time frame was applied systematically to epidemiological and clinical studies assessing associations between nutritional factors and myopia outcomes. Earlier studies were included selectively to provide biological and mechanistic context where relevant.

The inclusion criteria comprised randomized controlled trials, cohort studies, cross-sectional studies, and case–control studies evaluating the relationship between dietary factors or serum nutrient levels and myopia incidence, progression, refractive error, or axial length. The exclusion criteria included editorials, conference abstracts, case reports, narrative reviews without original clinical data, and studies lacking relevant exposure or outcome measures.

The study selection process was conducted in two stages by three independent reviewers. First, duplicate records were identified and removed. Subsequently, titles and abstracts were screened for relevance. Full-text articles of potentially eligible studies were then assessed according to predefined eligibility criteria. Discrepancies were resolved through discussion.

**Figure 2:**
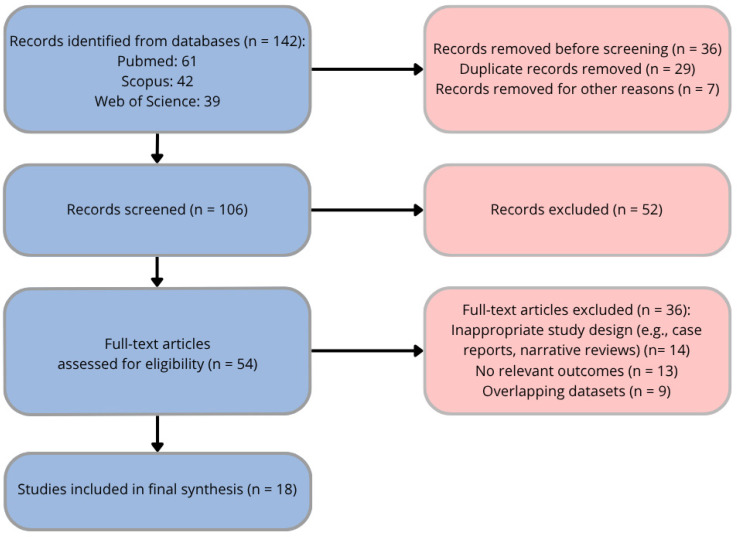
A flow diagram of the study selection process.

A total of 142 records were initially identified through database searching (PubMed: 61; Scopus: 42; Web of Science: 39). After the removal of 29 duplicate records and 7 records excluded for other reasons (e.g., inappropriate publication type), 106 titles and abstracts were screened. Of these, 52 records were excluded due to lack of relevance to the topic. The full texts of 54 articles were assessed for eligibility, of which 36 were excluded for the following reasons: inappropriate study design (*n* = 14), lack of relevant outcomes or insufficient data on nutritional exposure (*n* = 13), and overlapping datasets (*n* = 9). Ultimately, 18 studies were included in the final synthesis. The study selection process is summarized in Figure 2.

Data from the included studies were extracted descriptively, focusing on study design, geographic location, population characteristics, type of nutritional exposure (dietary intake or serum levels), and ophthalmic outcomes, including refractive error, axial length, and myopia prevalence or progression.

The methodological quality of the included studies was assessed narratively. No formal risk-of-bias tool was applied due to the narrative design of this review, as the aim was to provide a broad, integrative overview of the available evidence rather than a quantitative synthesis; however, key domains such as study design, control of confounding factors (e.g., time spent outdoors, parental myopia), exposure assessment, and outcome measurement reliability were critically considered.

Due to heterogeneity in study design, exposure definitions, and outcome reporting, findings were synthesized qualitatively rather than quantitatively. The outcomes of interest included myopia incidence, progression, refractive error, and axial elongation. Given the narrative nature of this review, a formal PRISMA-guided systematic review protocol and registration were not applied. No new human or animal research was conducted; therefore, ethical approval was not required (Figure 2).

## 3. Vitamin D

### 3.1. Biological Background of Vitamin D in Ocular Growth

Vitamin D is a secosteroid hormone well known for controlling calcium and phosphate balance in the body, and has been proposed to play a role in ocular tissues. However, its relevance for human eye growth remains uncertain. The vitamin D receptor (VDR) is present in many eye cells, including corneal endothelial cells, the nonpigmented ciliary body epithelium, and retinal pigment epithelial cells, indicating potential responsiveness to vitamin D, although its significance for refractive development is not established. Human eye barrier cells, like scleral fibroblasts, corneal cells, and retinal pigment epithelial cells express VDR and enzymes required to convert inactive vitamin D into its active form, 1,25-dihydroxyvitamin D_3_ [9].

Experimental data from animal models of myopia support functional evidence for the relevance of vitamin D signaling in eye growth regulation. In a mouse model, administration of a vitamin D_3_ analogue (calcipotriol) attenuated myopia progression. It also prevented the decrease in scleral VDR and helped maintain normal levels of type I collagen in the sclera [14]. While informative, these findings are derived from controlled experimental conditions and their translation to human physiology remains uncertain. Similarly, VDR agonists such as calcitriol have been shown to alter angiogenesis and regulate molecules such as microRNA-21 in vertebrate eye development models, suggesting potential effects on ocular structure and vascular development, although their clinical relevance is unclear [15].

Vitamin D signaling has also been implicated in immunomodulation and epithelial barrier function. Ocular epithelial cells can locally synthesize active vitamin D, which may contribute to local immune regulation and barrier integrity [9].

Retinal physiology may also be influenced by vitamin D: active vitamin D metabolites have been detected in aqueous and tear fluids, and UV-B exposure in vitro promotes synthesis of vitamin D_3_ metabolites in corneal epithelial cells, indicating that sunlight-dependent mechanisms may directly affect local ocular vitamin D levels [10]. Nevertheless, these findings remain largely experimental and do not establish causality in myopia development.

Taken together, these findings suggest a potential biological role of vitamin D in ocular tissues (Table 1). Although ocular tissues demonstrate responsiveness to vitamin D signaling, the evidence is predominantly preclinical and its translational relevance to human myopia remains uncertain.

### 3.2. Children and Adolescents

Large population-based studies consistently report lower serum 25-hydroxyvitamin D [25(OH)D] levels in myopic children and adolescents compared with non-myopic individuals [16].

Cross-sectional analyses demonstrate significant inverse, dose–response associations between serum 25(OH)D concentration and myopia prevalence [16]. However, the cross-sectional nature of these data precludes causal inference. In Chinese children, vitamin D deficiency was highly prevalent and, after controlling for gender, parental myopia, after-school class attendance, and outdoor activities, was associated with more than a twofold increased risk of moderate and high myopia [8]. Similarly, Korean adolescents with the lowest 25(OH)D levels exhibited the highest prevalence of myopia, particularly high myopia, supporting a severity-dependent association [17]. These findings are consistent but remain observational in nature.

Structural ocular parameters provide additional insight into the relationship between vitamin D and myopia. Lower 25(OH)D levels have been associated with longer axial length, a key determinant of myopia [1]. Each 25 nmol/L increase in 25(OH)D was linked to a 35% reduction in myopia odds. Although this may suggest an association beyond sunlight exposure alone, residual confounding cannot be excluded. Vitamin D may still act as a proxy for unmeasured aspects of light exposure or lifestyle.

Reverse causation should also be considered, as myopic children may spend less time outdoors, leading to lower vitamin D levels. Longitudinal data further weaken a causal interpretation.

A prospective birth cohort study in Taiwanese children found no association between serum 25-hydroxyvitamin D [25(OH)D] concentrations measured at birth and during early childhood (at 1, 3, and 5 years of age) and subsequent myopia development or axial length. These findings challenge the assumption of a direct, causal role of vitamin D in refractive development and instead suggest that previously reported associations may be confounded by environmental exposures. In particular, factors such as high educational intensity, near-work demands, and limited outdoor light exposure—especially prevalent in East Asian populations—are likely to exert a stronger influence on ocular growth trajectories. Moreover, the lack of association across multiple early-life time points weakens the hypothesis of a critical developmental window for vitamin D in myopia pathogenesis [2] (Table 2).

### 3.3. Young Adults

Evidence in young adults mirrors pediatric findings but remains observational. In the Raine Study, participants with serum 25(OH)D_3_ concentrations below 50 nmol/L exhibited more than twice the odds of myopia compared with those with higher levels. This association remained significant after adjustment for parental myopia, ethnicity, education level, and sun exposure, suggesting a potentially independent relationship between vitamin D status and refractive error [18]. Comparable findings from adult NHANES data showed lower mean serum vitamin D concentrations among myopic individuals relative to non-myopic participants [26]. Meta-analyses suggest association between higher serum 25(OH)D levels and reduced odds of myopia in young adults, with stronger associations observed for vitamin D_3_ than for total vitamin D [32]. Nevertheless, these results should be interpreted cautiously due to the predominance of observational data.

### 3.4. Prenatal Vitamin D Status

Prenatal development is a critical period for ocular and neural maturation. Maternal vitamin D status has been linked to neurodevelopmental outcomes, including visual-motor performance [33,34]. Studies on prenatal vitamin D and postnatal refractive development suggest that maternal vitamin D status influences systemic developmental pathways that may overlap with mechanisms involved in eye growth. However, direct evidence linking prenatal vitamin D to refractive outcomes is limited. Longitudinal and case–control studies report no significant association between prenatal or neonatal vitamin D status and later myopia, indicating that prenatal vitamin D is unlikely to be a strong independent predictor of myopia [2,35].

In contrast, Tong et al. suggest that maternal vitamin D deficiency may interact with prenatal environmental exposures, such as arsenic, to influence offspring axial length and myopia risk, particularly in male children. In the study, elevated arsenic concentrations in umbilical cord blood were associated with longer axial length and increased odds of myopia in primary school children. The association between prenatal arsenic exposure and axial elongation was significantly stronger among children whose mothers had vitamin D deficiency during pregnancy. Children born to vitamin D-deficient mothers exhibited nearly double the risk of developing myopia compared with those whose mothers had sufficient vitamin D levels [36]. These findings support a potential modifying rather than direct causal role of vitamin D.

### 3.5. Time Spent Outdoors and Sunlight Exposure

Time spent outdoors is one of the most consistently identified protective factors against myopia in children and adolescents [3]. Sunlight exposure stimulates vitamin D_3_ synthesis, linking outdoor activity with serum vitamin D levels [37].

Observational studies report that associations between vitamin D and myopia persist after adjustment for outdoor time [18,34,36]. Meta-analytic evidence supports this observation, indicating that higher serum 25(OH)D and particularly vitamin D_3_ levels are associated with lower myopia risk independent of sunlight exposure. However, these findings should be interpreted cautiously, as residual confounding is likely. Accurately quantifying outdoor exposure remains methodologically challenging, with most studies relying on self-reported data that do not capture light intensity, timing, or cumulative exposure patterns. Consequently, statistical adjustment may not fully disentangle the complex interplay between light exposure, behavior, and metabolic factors.

Longitudinal data reinforce the central role of outdoor exposure. The study by Lingham et al. demonstrated that greater time spent outdoors during childhood was associated with a reduced risk of myopia in early adulthood, independent of confounders such as parental myopia and educational exposure [3]. Complementary findings indicate that lower serum 25(OH)D trajectories during adolescence-likely reflecting reduced outdoor activity-are associated with an increased risk of myopia by age 20 [4].

Importantly, more controlled clinical data provide further insight into this relationship. In a cross-sectional study of school-aged children born preterm, greater time spent outdoors was associated with significantly lower odds of myopia even after adjustment for key confounders, including near-work, parental myopia, and serum 25(OH)D levels (OR 0.13 per additional hour/day). In contrast, serum vitamin D concentrations showed no association with refractive error or spherical equivalent, despite a high prevalence of vitamin D insufficiency in the cohort. These findings underscore that outdoor exposure itself- rather than vitamin D status- may be the dominant factor influencing refractive development [24].

### 3.6. Genetic Evidence

Additional evidence from genetic studies further challenges the hypothesis of a direct causal role for vitamin D.

Analyses of polymorphisms in genes involved in the vitamin D pathway, including the vitamin D receptor (VDR), have not demonstrated a consistent genetic association with myopia [32]. This lack of genetic association further supports the interpretation that vitamin D may primarily act as a biomarker of outdoor exposure or a modifier of environmental effects rather than a direct causal factor.

This interpretation is supported by large genome-wide association studies, which have identified multiple loci linked to refractive error, predominantly related to retinal signaling, extracellular matrix remodeling, and ocular development, without implicating vitamin D metabolic or signaling pathways. While these data do not exclude a modest or indirect contribution, they suggest that vitamin D is unlikely to play a primary causal role in myopia development [38].

Taken together, the available evidence supports the interpretation that vitamin D is more likely a surrogate marker of outdoor exposure or a modifier of environmental effects rather than an independent determinant of myopia development.

## 4. The Impact of Other Selected Nutrients on the Development of Nearsightedness

### 4.1. Vitamin A

Vitamin A is widely recognized as a crucial, fat-soluble micronutrient essential for normal growth, immune competence, and vision. Its derivative, 11-cis-retinal, is a prerequisite for rhodopsin formation in rod photoreceptors [39]. Furthermore, this nutrient and its active metabolite, retinoic acid, regulate gene expression to control epithelial integrity and tissue differentiation [40].

Despite its physiological importance, hypovitaminosis A remains a major global public health challenge [41]. It disproportionately affects children under five years of age as well as pregnant and lactating women, particularly in low- and middle-income regions, including sub-Saharan Africa and South Asia [42,43].

Dietary intake is derived from two main forms: preformed retinol, found in animal products such as beef liver and full-fat dairy, and provitamin A carotenoids, abundant in plant sources such as sweet potatoes, carrots, pumpkins, and dark green leafy vegetables [39]. In high-risk settings, the WHO recommends periodic high-dose supplementation for children aged 6–59 months at intervals of four to six months [41]. Both randomized trials and observational data confirm that such interventions in deprived populations significantly reduce child mortality and the incidence of clinical eye diseases [42].

At the molecular level, this micronutrient provides the 11-cis-retinal required for phototransduction and low-light vision [39]. Simultaneously, retinoic acid modulates immune cell differentiation and mucosal immunity [44].

Consequently, clinical manifestations of deficiency typically begin with night blindness and xerophthalmia. Progressive depletion leads to Bitot’s spots and corneal ulceration, potentially culminating in permanent vision loss [41]. Moreover, this nutritional inadequacy exacerbates susceptibility to severe infections, including measles, diarrhoea, and pneumonia [42].

Given these ocular and molecular functions, vitamin A status has been hypothesized to influence refractive development. A large population-based investigation in Korea demonstrated a statistically significant correlation between higher serum levels of vitamin A and a reduced prevalence of myopia. Individuals in the highest quartiles of serum vitamin A had lower odds of both general and severe refractive errors. These associations remained significant after adjusting for potential confounders such as age, sex, and systemic health factors. These findings suggest that higher systemic vitamin A status is associated with a reduced prevalence of myopia in adults [11].

By contrast, longitudinal data following individuals from adolescence into early adulthood presents a different perspective. Initially, unadjusted data suggested a protective effect against myopia, but this link lost statistical significance after adjusting for variables such as time spent outdoors, education level, and parental history. These findings imply that total dietary consumption alone may not be a primary determinant of refractive outcomes in young adults [21].

However, biochemical data from pediatric populations further highlight the complexity of this relationship. Children with advanced myopia exhibit significantly lower plasma retinol concentrations compared to healthy controls. Notably, circulating levels correlate negatively with ocular axial length, suggesting a potential link between poor nutritional status and the excessive eye elongation characteristic of this condition. These results may reflect underlying physiological mechanisms, as retinoic acid modulates gene expression during eye development and may influence scleral remodeling [27].

Overall, the current evidence regarding this micronutrient and refractive health remains inconclusive. Discrepancies between analyses based on blood biomarkers, dietary intake, and clinical measurements prevent the establishment of a clear causal link. While adequate intake is undeniably essential for ocular physiology, its specific role in preventing or retarding the progression of visual impairment remains uncertain and warrants further research.

### 4.2. Microelements: Zinc and Selenium

Zinc and selenium are essential trace elements required for normal growth, immune function, and antioxidant defence [45]. Zinc is particularly concentrated in the retina, where it supports photoreceptor function and synaptic transmission [46]. Selenium, meanwhile, is an integral component of selenoproteins, including glutathione peroxidases and deiodinases, which protect cells against oxidative damage and regulate thyroid hormone metabolism [47]. Owing to their roles in ocular antioxidant defence and immune regulation, these micronutrients are biologically relevant to eye health and may potentially influence refractive development [48].

Epidemiologically, zinc and selenium deficiencies remain common in populations with limited dietary diversity or low soil mineral content, affecting children and other vulnerable groups worldwide [12,13,49,50]. Nutritional inadequacies have also been reported in European populations, including Poland, as well as in clinical groups such as preterm infants or children following restrictive diets [13]. However, despite this well-documented prevalence, the direct relationship between these deficiencies and refractive errors remains insufficiently characterised, as most studies have not assessed ocular growth parameters such as axial length or refractive progression.

From a nutritional perspective, zinc deficiency can generally be prevented through balanced diets containing animal-derived foods with high bioavailability [51]. Selenium intake is similarly supported by protein-rich foods, including fish and meat, with some foods such as Brazil nuts representing concentrated sources [52]. Typical mixed diets including those introduced during complementary feeding are generally sufficient to meet selenium requirements in children [13]. When dietary intake is inadequate, supplementation may be considered [51,52]. Nevertheless, supplementation aimed specifically at preventing refractive errors cannot currently be recommended because of insufficient clinical evidence.

At the physiological level, zinc deficiency in children compromises development and impairs wound healing and sensory perception [53]. On the cellular level, zinc contributes to the reduction in oxidative stress and inflammation through modulation of signaling pathways and antioxidant enzymes, with particularly important roles in the retina, where it supports synaptic transmission and photoreceptor integrity [47]. These retinal functions are of particular interest in refractive research, as experimental models of myopia indicate that altered retinal neurotransmission may influence scleral remodeling and ocular elongation. Similarly, selenium functions primarily as a component of protective selenoenzymes, including glutathione peroxidases [50], and deficiency of these enzymes increases susceptibility to oxidative damage in tissues [19,54]. Although oxidative stress has been proposed as one of the mechanisms contributing to myopic progression, direct clinical evidence linking selenium deficiency to measurable refractive changes remains inconclusive.

Research regarding trace minerals in myopic children remains inconsistent. Fedor et al. reported that children with myopia exhibited lower concentrations of zinc and selenium, accompanied by a significantly higher copper-to-zinc ratio. However, the levels of copper and manganese were comparable between the groups. The authors suggested that deficits in zinc and selenium might contribute to the progression of myopia, given their antioxidant properties and protective roles in the eye [20]. Considering zinc’s high retinal concentration and selenium’s importance for ocular enzymes, the study suggests that the potential benefits of supplementation require further investigation [19,46].

On the other hand, conflicting evidence exists. Burke et al. conducted a large population-based survey in the USA and found no significant association between zinc intake and myopia in children and adolescents aged 12 to 19 years [20].

In summary, although zinc and selenium play important roles in ocular biology and oxidative stress regulation, current human data do not establish a clear causal relationship between these trace elements and the risk of myopia.

### 4.3. Omega-3 Polyunsaturated Fatty Acids (PUFAs)

The development of myopia is primarily driven by excessive axial elongation of the eye, a process governed by complex interactions among retinal signaling pathways, choroidal blood flow, and scleral remodeling, which may be influenced by the availability of omega-3 polyunsaturated fatty acids (PUFAs). Omega-3 PUFAs have been studied as potential protective factors against myopia, largely on the basis of their anti-inflammatory and vasodilatory properties. One cohort study found that children with high dietary eicosapentaenoic acid (EPA) intake (≥11 mg per 1000 kcal) had significantly lower odds of high myopia (OR ≈ 0.39, 95% CI: 0.18–0.85) than those with lower EPA intake [25].

Moreover, a large study reported that higher genetically predicted plasma levels of total omega-3 PUFAs, docosahexaenoic acid (DHA) and EPA were each inversely associated with myopia risk. Increased omega-3 PUFAs exposure, particularly DHA, was causally associated with a shift in refractive error toward hyperopia, supporting Mendelian randomization evidence indicating that omega-3 PUFAs may protect against myopia by modulating choroidal blood perfusion involved in ocular growth regulation [29]. Consistent with these findings, observational data from Asian children indicate that those with the lowest omega-3 intake had longer axial lengths and more myopic refraction than children with higher omega-3 intake [28]. A study published in 2025 demonstrated that low omega-3 PUFA intake was significantly associated with myopia, and the authors concluded that higher dietary omega-3 may protect against myopia development in children [30].

Importantly, the potential protective role of omega-3 PUFAs should be interpreted in the context of widespread suboptimal intake at the population level. Global assessments of long-chain omega-3 PUFA status, commonly measured using the Omega-3 Index (EPA + DHA in erythrocyte membranes), indicate that most studied populations worldwide fall into low or very low ranges, well below levels considered optimal for health [55]. This suggests that a substantial proportion of children may be exposed to a nutritional environment that is not conducive to optimal ocular development. In this context, population-level deficiency or suboptimal status of omega-3 PUFAs may contribute susceptibility to dysregulated ocular growth and myopia development, thereby increasing the relevance of dietary omega-3 intake as a potentially modifiable protective factor.

Taken together, the available epidemiological, genetic, and observational evidence suggests a plausible protective role of omega-3 PUFAs against myopia development. However, despite the consistency of these findings, the precise mechanisms underlying this association, as well as the magnitude of the potential protective effect in different populations, remain to be fully elucidated. Further research is crucial to clarify the full mechanisms underlying the influence of omega-3 PUFAs on myopia development.

### 4.4. Refined Carbohydrates

High consumption of refined carbohydrates has been reported to be associated with an increased risk of myopia in epidemiological studies. Data from French cohorts suggest that higher intake of processed carbohydrates may be linked to a greater probability of myopia, with sex-specific differences. In children, frequent consumption of refined carbohydrates was associated with a significantly higher risk of myopia in girls, while an inverse association was observed in boys [22]. Interestingly, analysis of the other cohort suggested that a higher dietary glycemic load, reflecting increased carbohydrate intake, was associated with a greater prevalence of myopia in adult men, but not in women [31].

Beyond observational correlations, a biologically plausible mechanism has been hypothesized linking refined carbohydrate intake to myopia development. Diet rich in rapidly absorbed carbohydrates can induce significant spikes in insulin levels and, over time, contribute to chronic hyperinsulinemia. This phenomenon may promote metabolic dysregulation and insulin resistance in peripheral tissues [56]. It has been proposed that chronic hyperinsulinemia may elevate insulin-like growth factor (IGF-1) activity in scleral fibroblasts, potentially enhancing proliferation and extracellular matrix remodeling of the sclera. These processes have been implicated in axial elongation characteristic of myopia.

Insulin-related growth factor pathways have been shown in experimental animal models to modulate eye growth and ocular dimensions, with IGF-1 and related factors associated with elongation of the vitreous chamber and refractive shifts toward myopia. However, these findings do not establish causality in humans [57]. Supporting this limitation, clinical evidence regarding the role of IGF-1 in humans remains inconsistent. For instance, an analysis of a large Japanese cohort found no significant association between serum IGF-1 levels and high myopia, suggesting that the relationship between circulating IGF-1 and myopia pathogenesis may be more complex or population-dependent than experimental models imply [58].

Taken together, these findings suggest a possible association between high refined carbohydrate intake and myopia risk, whereas the proposed metabolic mechanisms involving hyperinsulinemia and scleral remodeling remain hypothetical and warrant further investigation [Table 3].

## 5. Conclusions

Myopia development appears to result from a multifactorial interaction between environmental, metabolic, and, potentially, nutritional influences. Observational studies consistently report associations between higher serum 25(OH)D levels and more favorable myopia-related outcomes. However, current evidence indicates that vitamin D is more likely to reflect outdoor exposure than to act as an independent causal factor. Accordingly, assessment of vitamin D status may be relevant in the context of general health, whereas its role in routine myopia prevention or control remains unsupported by current evidence.

Among the evaluated dietary components, omega-3 fatty acids appear to be the most promising modifiable nutritional factor. Their potential relevance is supported by biological plausibility, observational data, and emerging genetic evidence, although the magnitude of their effect and clinical significance require further clarification. High intake of refined carbohydrates has also been associated with myopia in some studies, but the proposed insulin-mediated mechanisms involving scleral remodeling remains hypothetical. In contrast, the roles of vitamin A, zinc, and selenium in refractive development remain inconclusive, despite their established importance for retinal health and antioxidant defense.

Overall, the current literature does not support specific nutritional interventions as standalone strategies for myopia control. Nutrition is better interpreted within a broader behavioral and environmental framework, particularly in relation to outdoor exposure, near-work patterns, and overall metabolic health. Further longitudinal and interventional studies using objective exposure assessment and adequate control of confounders are needed to determine whether nutritional factors exert independent and clinically meaningful effects on axial elongation.

## Figures and Tables

**Table 1:** Vitamin D signaling in ocular tissues and its potential relevance to myopia.

Ocular Structure	VDR Expression/Local Vitamin D Activation	Biological Effect	Relevance to Myopia
Corneal endothelium	+/+	Barrier function	Environmental response
Corneal epithelium	+/UV-B-induced	Local synthesis and immunomodulation	Link to light exposure
Nonpigmented ciliary epithelium	+/+	Intraocular regulation	Indirect growth role
Retinal pigment epithelium	+/+	Angiogenesis, signaling	Retinal development
Scleral fibroblasts	+/+	Collagen synthesis	Axial elongation
Aqueous/tear fluid	−/metabolites	Active forms detected	Local light-dependent metabolism

Abbreviations: VDR, vitamin D receptor; UV-B, ultraviolet B radiation. This table summarizes experimental and observational evidence on vitamin D signaling in ocular tissues. Most findings are derived from preclinical studies, and their direct relevance to human myopia development remains uncertain.

**Table 2:** Summary of clinical and epidemiological studies evaluating the association between nutritional factors and myopia (2014–2025). Abbreviations: 25(OH)D, 25-hydroxyvitamin D; PUFA, polyunsaturated fatty acids; EPA, eicosapentaenoic acid; DHA, docosahexaenoic acid.

Author	Year	Country	Study Design	Population	Exposure	Definition	Outcome	Findings
Choi [17]	2014	Korea	Cross-sectional	Adolescents	Vitamin D	Serum 25(OH)D levels	Myopia severity	Stronger association in high myopia
Yazar [18]	2014	Australia	Cohort	Young adults	Vitamin D	Serum 25(OH)D levels	Myopia	Lower vitamin D associated with higher odds
Fedor [19]	2017	Poland	Case–control	Children	Zinc, Selenium	Serum trace element levels	Myopia	Lower zinc and selenium in myopic group
Burke [20]	2019	USA	Cross-sectional	Adolescents	Zinc	Dietary zinc intake	Myopia	No significant association
Ng [21]	2020	Australia	Cohort	Young adults	Vitamin A	Dietary intake	Myopia	No independent association after adjustment
Berticat [22]	2020	France	Cross-sectional	Children	Refined carbohydrates	Dietary intake frequency	Myopia	Higher risk observed (sex-specific)
Gao [23]	2021	China	Cross-sectional	Children	Vitamin D	Serum 25(OH)D concentration	Myopia	Lower levels observed in myopic individuals
Chou [24]	2021	Taiwan	Cross-sectional	Children (preterm)	Vitamin D/Outdoor exposure	Serum 25(OH)D and time outdoors	Myopia	Outdoor time significant; vitamin D not associated
Zhou [25]	2023	USA	Cross-sectional	Children	Omega-3 PUFAs	Dietary EPA intake	High myopia	Protective association
Tao [16]	2024	China	Cross-sectional	Children/adolescents	Vitamin D	Serum 25(OH)D levels	Myopia prevalence	Inverse dose–response association
Wolf [26]	2024	USA	Cross-sectional	Adults	Vitamin D	Serum vitamin D concentration	Myopia	Lower levels in myopic participants
Mikoluc [27]	2024	Poland	Case–control	Children	Vitamin A	Plasma retinol levels	Axial length/high myopia	Lower levels associated with high myopia
Xue [28]	2024	Multi-national	Observational	General population	Omega-3 PUFAs	Plasma PUFA levels	Refractive error	Protective trend observed
Li [2]	2025	Taiwan	Prospective cohort	Children	Vitamin D	Longitudinal serum 25(OH)D (birth–childhood)	Myopia development	No significant association
Lee & Jee [11]	2025	Korea	Cross-sectional	Adults	Vitamin A	Serum vitamin A levels	Myopia prevalence	Higher levels associated with lower risk
Lu [29]	2025	Multi-national	Mendelian randomization	General population	Omega-3 PUFAs	Genetically predicted PUFA levels	Myopia risk	Inverse causal association suggested
Zhang [30]	2025	Hong Kong	Cohort	Children	Omega-3 PUFAs	Dietary intake	Axial length	Reduced axial elongation
Berticat [31]	2025	France	Cohort	Adults	Glycemic load	Dietary glycemic load	Myopia prevalence	Association observed in men

**Table 3:** Selected nutrients and their potential role in myopia development. Abbreviations: PUFAs, polyunsaturated fatty acids; IGF-1, Insulin-like Growth Factor-1.

Nutrient	Mechanism	Evidence	Conclusion
Vitamin A	Retinoic signaling; phototransduction	Inconsistent; biomarker vs intake mismatch	No casual evidence
Zinc	Retinal function; antioxidant	Conflicting; weak epidemiology	Unclear
Selenium	Antioxidant enzymes	Inconsistent	No evidence
Omega-3 PUFAs	Anti inflammatory; ocular blood flow	Consistent observational + genetic signals	Probable protective
Refined carbohydrates	Insulin/IGF-1 axis; scleral remodeling (hypothetical)	Inconsistent	Possible risk (unproven)

## Data Availability

No new data were created or analyzed in this study.
